# Helikite aerial sensing system: An innovative approach for monitoring construction sites

**DOI:** 10.1371/journal.pone.0352261

**Published:** 2026-07-16

**Authors:** Cheng Fei, Hui Liu, Zhuanyun Yang, Xiuchun Ji, Runhao Guo

**Affiliations:** 1 Department of Civil Engineering, Sichuan College of Architectural Technology, Chengdu, China; 2 School of Railway Transportation, Guangzhou Railway Polytechnic, Guangzhou, China; King Fahd University of Petroleum & Minerals, SAUDI ARABIA

## Abstract

This study proposes an innovative Helikite-based aerial sensing system as a reliable aerial monitoring solution for construction sites. Construction site monitoring requires real-time, unobstructed, and long-duration coverage to ensure worker safety and project efficiency, but this remains challenging due to the limitations of existing methods. Key factors contributing to this challenge include: 1) fixed cameras are prone to occlusion and have a restricted range in dynamic construction environments; 2) UAVs are limited by short battery life, limited wind tolerance, and high maintenance costs; and 3) there is a lack of bird’s-eye-view datasets. This study hypothesizes that the Helikite aerial sensing system can operate stably across the wind speed range typical of construction site operations. The methodology involves three core steps: 1) designing a wind-resistant Helikite-based system capable of long-duration operation; 2) constructing a specialized dataset—comprising 1,000 original bird’s-eye-view images of construction sites expanded to 3,000 through data augmentation—to address the data gap; and 3) validating the system using the YOLO-v5 model for worker recognition on an independent test set of 100 images. Experimental results show the YOLO-v5 model achieved 0.9827 accuracy, 0.9850 precision, 0.9920 recall, and 0.9820 F1-score on the validation set, confirming the high quality of Helikite-captured data and the system’s effectiveness.

## 1. Introduction

Real-time monitoring on construction sites is essential for ensuring worker safety, maintaining project schedules, and achieving quality control [1–3]. Construction sites continuously generate vast quantities of real-time data that must be captured and processed to support safety management and operational decision-making. Continuous visual data captured by fixed cameras provide critical information about on-site resources, including worker activities and equipment movements [4]. Subsequently, computer vision techniques have been increasingly adopted for localizing construction resources—such as workers, excavators, and cranes—as well as for analyzing spatial utilization patterns on site [5] and predicting proximity conflicts among mobile equipment [6].

With the advent of deep learning techniques, motion recognition, and tracking, extracting specific body skeletons from images or videos has become possible. Several studies have applied deep learning methods to monitor construction activities [7–9]. Risky behaviors—including falling from heights, failure to wear hard hats or personal protective equipment (PPE), and improper handling of hazardous materials—can now be identified by cameras equipped with such deep-learning-based innovations. Given the critical importance of real-time monitoring on construction sites, selecting the appropriate monitoring equipment is a prerequisite for effective information collection and processing.

The remainder of this paper is organized as follows. Section 2 reviews existing monitoring methods—fixed cameras, UAVs, and tethered balloons—and highlights their limitations. Section 3 presents the architecture of the proposed Helikite-based system, covering its mechanical design, data acquisition, transmission, and processing modules. Section 4 describes the experimental setup, including data collection, augmentation, and model training. Section 5 reports the experimental results and performance metrics, followed by a discussion of the system’s effectiveness, challenges, and advantages over UAV-based approaches in Section 6. Section 7 outlines the limitations of the current work and suggests directions for future research. Finally, Section 8 concludes the paper with a summary of contributions and potential impact.

## 2. Related work

### 2.1 The fixed camera

Although previous studies have confirmed that computer vision can provide reliable detection capabilities for construction sites, camera placement remains a significant challenge due to the dynamic and complex nature of these environments [4]. Achieving full site coverage requires careful determination of the number, type, locations, and orientations of fixed cameras. However, the conventional manual placement process is both time-consuming and labor-intensive, often resulting in suboptimal configurations that compromise monitoring performance. Traditionally, cameras are mounted on existing structures, trees, or other elevated positions to achieve high-altitude monitoring; however, the monitoring range is severely constrained by the fixed installation height and position [10,11]. Once the camera installation position fails to meet requirements, re-planning and re-installing the layout will consume substantial resources. Consequently, considerable research efforts have been directed toward determining the optimal sensor network configuration under given constraints [12]. However, they have primarily focused on camera placement in relatively organized environments for monitoring [4]. It has further been noted that fixed cameras on dynamic construction sites often face sudden occlusion by moving equipment, leading to intermittent monitoring blind spots [13]. While subsequent efforts optimized fixed camera networks through genetic algorithms, their solutions still failed to adapt to temporary construction layouts [14].

### 2.2 Unmanned aerial vehicles

Some studies have attempted to use mobile detection equipment such as UAVs (Unmanned Aerial Vehicles) or robots for efficient construction site monitoring, with UAVs being the mainstream choice for medium and low-altitude mobile monitoring [15,16]. The flight of a UAV is sustained by generating thrust and lift to overcome gravity, a process that fundamentally limits its onboard energy budget [17]. Previous research provided a comprehensive review of the latest advancements in UAV monitoring and inspection within the construction industry [18]. The authors analyzed various types of UAVs and sensors along with their applications, highlighted both technological innovations and associated challenges, and proposed future research directions that underscore the advantages of UAVs in construction inspection. Other studies developed an automated method for recognizing construction areas by leveraging deep learning and time-lapse photography techniques [19]. Through parameter optimization, this technique efficiently identifies changing locations in time-series photographs of construction sites with high accuracy, holding promise for advancing intelligent construction technologies. Related work introduced an image-based automated approach that utilizes Convolutional Neural Networks (CNNs) to detect and locate construction defects [20]. The method employs Class Activation Mapping (CAM) for object localization and builds upon a pre-trained VGG-16 classifier, enabling real-time detection and localization on UAVs and mobile devices. Additional research explored the potential of Digital Twin (DT) technology in the construction sector, conducting a detailed analysis of the strengths and weaknesses of UAVs in Construction Informatics (CI) [21]. The author advocated the use of UAVs to track progress at various stages of construction.

UAVs can operate at varying altitudes and be equipped with different sensors to suit specific observation tasks on construction sites. However, UAVs suffer from short flight endurance due to their reliance on limited onboard battery capacity [22]. Current battery technology suffers from low energy density and long recharging times, which severely limit the flight endurance of UAVs [23]. A solar-assisted energy augmentation approach has been proposed to extend UAV flight time; however, it remains insufficient for all-day continuous monitoring [24]. Although aerodynamic improvements have enhanced UAV wind resistance to some extent, most models still struggle to maintain stable flight under windy conditions, restricting their applicability on exposed construction sites [25]. In summary, UAV-based monitoring approaches face three major limitations. First, the difficulty of providing a continuous energy supply prevents UAVs from remaining airborne for extended periods. Second, UAVs are highly susceptible to adverse weather conditions such as rain and strong winds, which severely degrade their operational reliability [26]. Third, the maintenance costs of UAV systems remain relatively high, especially in the harsh environments typical of construction sites [27].

**Fig 1:**
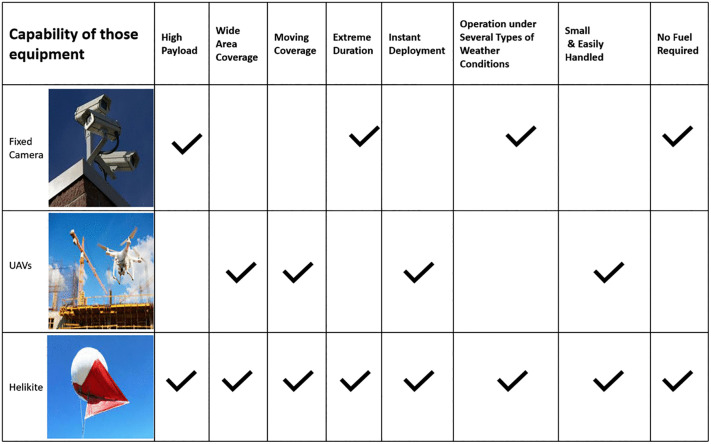
Comparison of different monitoring methods.

In summary, although UAVs offer a highly mobile platform for construction site monitoring, their limited endurance, vulnerability to wind, and high operational costs restrict their suitability for scenarios that demand persistent, long-duration observation. The Helikite system presented in this study aims to address these limitations. As illustrated in Fig 1, by combining helium buoyancy with kite aerodynamics, the Helikite offers distinct advantages in endurance, wind resistance, and cost-effectiveness, making it a complementary solution for persistent, low-altitude monitoring at a fixed point.

### 2.3 The tethered balloon-Helikite

In contrast to UAVs, which require a continuous power supply for active propulsion, unpowered lighter-than-air aerostats have attracted considerable research interest. An aerostat is a lighter-than-air platform that achieves buoyancy through a lifting gas rather than aerodynamic lift, and is commonly employed for high-altitude missions. A tethered balloon is a type of aerostat that remains aloft solely through buoyancy, without requiring onboard propulsion [28]. Tethered balloons offer several advantages: extended airborne endurance, substantial payload capacity, wide-area observation coverage, ease of deployment, and low operational cost [29,30]. The tethered balloon remains aloft by being filled with a lighter-than-air gas and is controlled via tethering cables anchored to the ground [31]. The detection range of the tethered balloon monitoring system is broad, and it can detect low-altitude targets effectively [32].

Tethered balloons can sustain high-altitude monitoring for extended periods without an onboard energy supply and can carry substantial payloads [33]. A tethered balloon can be stationed at a fixed point in the air by means of a tethering cable [34]. A gas-tight tethered balloon can remain airborne continuously for several days. Compared with UAVs, tethered balloons exhibit a lower probability of in-flight damage and higher operational survivability. Using a tethered balloon as a sensor platform is cost-effective, with operational costs approximately 30% lower than those of comparable UAV-based systems [35,36].

However, most of the research about tethered balloons is focused on the design of a stable platform [30,31,37]. Recent studies indicate that traditional tethered balloons may exhibit position drift in wind speeds, affecting data consistency [38]. In contrast, Helikite’s integrated helium balloon and kite design reduces such drift under the same conditions, owing to its aerodynamic stability [39]. A typical tethered-balloon observation system comprises three main components: the tethered balloon itself, a stabilized platform suspended beneath it, and an optical camera mounted on the stabilized platform [35]. These balloons can carry integrated optical sensor payloads aloft, enabling long-term, long-distance monitoring and detection. Because tethered balloons are susceptible to wind-induced oscillations, a stabilized platform is required beneath the balloon to dampen camera vibrations and ensure high-quality data acquisition [31,40]. However, a stable platform is relatively expensive, complex, and not flexible. Moreover, the dead weight of the stabilized platform necessitates a larger tethered balloon to generate sufficient buoyancy to offset the additional load. Although the giant tethered balloon has great buoyancy, its volume limits its use in construction site monitoring. Construction sites, with their densely packed temporary structures and evolving layouts, require monitoring equipment that is compact, flexible, and easily deployable.

A Helikite is a tethered balloon that combines a helium balloon and a kite to form a single, aerodynamically sound tethered aircraft that exploits wind and helium for its lift [41]. The shape of the balloon typically resembles an oblate spheroid [42]. By integrating a helium balloon with kite wings, this innovative, lighter-than-air apparatus merges the optimal features of both designs. The balloon, inflated with helium, enables takeoff even in calm weather. Conversely, when wind is present, the kite elements prove indispensable: primarily, they elevate the entire system to heights exceeding those achieved solely by helium buoyancy. Additionally, as wind speed increases, the Helikite’s lifting capacity becomes stronger, with its maximum lift dependent on the Helikite’s dimensions. Finally, the wings serve to neutralize any instability inherent in balloons and blimps operated in windy environments, thereby ensuring the Helikite’s stability [34]. Researchers from the Center for Transportation Research and Education (CTRE) at Iowa State University have also documented the Helikite’s unique omnidirectional behavior. They evaluated and contrasted the photographic conditions produced by various aerial devices, namely a kite, blimp, Helikite, and balloon, across multiple wind scenarios [43]. Regarding its versatility, the Helikite surpasses similar devices, offering superior payload capacity for its size when juxtaposed with standard aerostats. Its resilience allows it to function effectively in stronger winds than conventional blimps or balloons. Furthermore, due to its ability to operate in challenging weather conditions such as rain, fog, or freezing temperatures, the Helikite exhibits greater operational flexibility than many unmanned aerial vehicles (UAVs). Helikites were recently successfully adopted for deploying flying base stations (BSs) at high altitudes [44].

For such reasons, the capabilities of the most suitable methods available for implementing optical sensing in construction sites are summarized in Fig 1.

Despite its potential, the application of Helikite technology in construction site monitoring remains unexplored. This paper seeks to bridge that gap by presenting an innovative, easily installable, and stable Helikite-based system tailored for construction site monitoring. This system promises a cost-effective and comprehensive approach to real-site supervision.

To the best of our knowledge, this study makes two primary contributions. First, we present a novel aerial dataset of construction sites captured via the Helikite system, comprising 3,000 bird’s-eye-view images (1,000 original and 2,000 augmented) that address the scarcity of overhead perspectives in existing benchmarks. Second, we have designed and implemented a Helikite-supported monitoring platform that integrates a wireless camera with a cloud communication module. The platform’s effectiveness is demonstrated through YOLO-v5-based worker detection, achieving 98.27% validation accuracy (Precision = 0.9850, Recall = 0.9920, F1-score = 0.9820). These advances establish a mobile, stable, and cost-effective solution for comprehensive construction-site supervision.

## 3. Methodology

### 3.1 Framework overview

**Fig 2:**
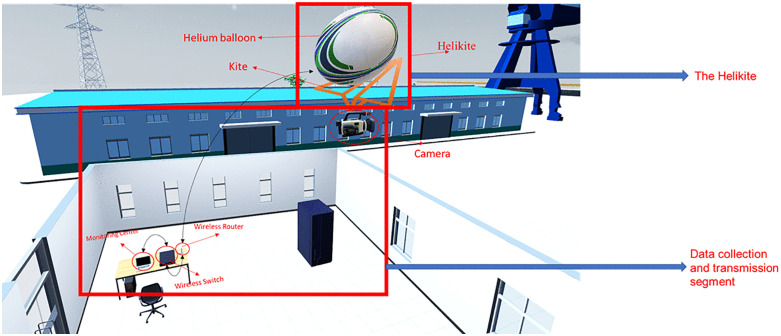
Pipeline of the presented framework.

This section introduces an innovative and robust autonomous monitoring system designed for long-term collection of real-time data at low and medium altitudes within construction sites. Comprising three integral components: the Helikite, the data acquisition and transmission module, and the data processing module, this system offers a comprehensive solution for construction site monitoring. The overall framework of this system is illustrated in Fig 2. The Helikite system combines the design of a helium balloon and a kite, forming an independent airborne sensor that is influenced by both wind force and helium lift. The system is capable of utilizing wind to gain altitude and maintain stability, making it effective even in windy environments. The main components include a helium balloon that serves as the primary source of buoyancy. The balloon is relatively insensitive to variations in temperature, pressure, and altitude, making it well-suited to the variable climatic conditions encountered on construction sites. The kite structure, consisting of a rigid carbon fiber spine, sails, and a keel, provides additional lift and stability. To meet the demands of construction environments, the keel and kite are constructed from lightweight carbon fiber, reducing overall weight and extending flight endurance. Furthermore, given the dusty conditions typical of construction sites, cameras with high waterproof and dustproof (IP) ratings were selected to ensure reliable operation in harsh environments. In contrast to a conventional tethered balloon, which is prone to positional shifts and instability due to wind forces, the Helikite exhibits superior wind resistance and stability. Its flight envelope remains stably centered above the release point, ensuring persistent coverage of the designated monitoring area.

The data acquisition and transmission module consists of a camera mounted at the base of the Helikite, a ground-based receiver, and a Wi-Fi communication link. This module enables real-time monitoring by providing video capture and wireless data transmission. The data processing module employs the YOLO-v5 algorithm to perform worker detection on the video footage captured by the Helikite-based system. The detection accuracy is then computed to evaluate the effectiveness and reliability of the system.

The proposed monitoring system provides a stable and practical solution for real-time data collection on construction sites, combining the aerodynamic advantages of the Helikite with advanced computer vision techniques.

### 3.2 Sub-systems description- Helikite

The Helikite, a pioneering hybrid of a kite and a balloon, introduces a novel concept to unmanned aerial vehicles. By integrating a helium-filled balloon with a kite structure, it forms a single tethered aircraft with high aerodynamic efficiency. The design leverages helium for buoyancy while harnessing wind power to enhance stability and generate supplementary lift.

The Helikite’s distinguishing features include a semi-rigid balloon inflated with helium and fortified with a rigid carbon fiber spine, carefully shaped for superior aerodynamics. Although the balloon typically assumes an oblate-spheroid shape, this geometry is not strictly imposed. Solid spars facilitate the attachment of payload equipment, such as video cameras, offering versatility in mission capabilities. Notably, in most windy scenarios, the kite’s aerodynamic lift surpasses the aerostatic lift provided by the helium.

**Fig 3:**
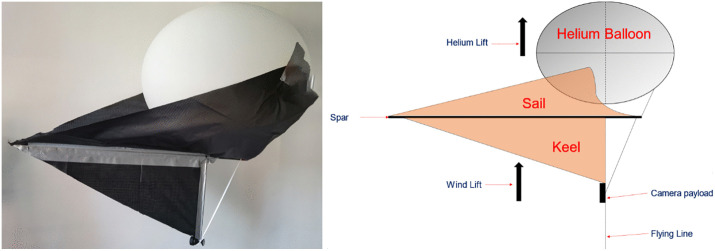
Main components of the Helikite system.

Engineered for all-weather, high-altitude missions, the Helikite features a robust and compact profile that enables operation in diverse climatic conditions at altitudes of up to 7,000 ft (approximately 2,134 m). In windy settings, both the primary aerodynamic and aerostatic lifts are situated at the front, while the spar weight and keel are positioned towards the rear. This strategic configuration ensures the Helikite’s stability, even in relatively strong winds. This inherent stability allows Helikites to operate with non-gyro-stabilized cameras, which demonstrates their practical adaptability. Consequently, Helikites are ideally suited for monitoring applications on construction sites, owing to their unique design and stability. The Helikite comprises several key components:

The helium balloon, remarkable for its minimal susceptibility to variations in temperature, pressure, or altitude, emerges as a distinctive feature of the Helikite’s aerodynamic architecture. Unlike conventional aerostats, this balloon serves a dual purpose: providing helium lift and contributing to wind lift. Meanwhile, the sail offers additional wind lift, leading to a steeper flight trajectory when exposed to wind forces. Moreover, it significantly enhances stability and damping capabilities. The keel, on the other hand, guarantees stability during high winds and aids in damping for the payloads. Its versatility is further highlighted by its ability to be folded away when the Helikite is stationary or being stored indoors or in a vehicle. The spar, spanning almost the entire length of the Helikite, constitutes its primary structure. This design aspect transforms the Helikite into a semi-rigid aerostat, equipping it to endure strong winds and turbulent air conditions. Ultimately, the flying line facilitates precise control over the tethered balloon’s altitude and operational range, showcasing the Helikite’s adaptability and maneuverability. The main components of the Helikite system can be seen in Figure 3.

The helium balloon is made of 0.12 mm-thick polyurethane film, with a helium retention rate of over 95% within 24 hours. The tether is a 3 mm diameter high-strength nylon rope with a tensile strength of 500 N, ensuring stability under wind loads. Operational uncertainties include helium leakage (≈0.5%/hour at 25℃, increasing to 1.2%/hour at 35℃) and keel vibration (amplitude <0.5° in winds <4 m/s), which are mitigated by regular helium supplementation and vibration-damping mounts.

### 3.3 Sub-systems description—Data collecting and transmission segment

#### 3.3.1 Wireless camera.

The wireless camera serves as the backbone of the surveillance system, functioning as an optical sensor that effortlessly converts light signals into electrical signals for seamless transmission. Known as a digital security camera or an Internet protocol camera, it operates by efficiently transmitting and receiving video content over an IP network. These cameras utilize a radio frequency (RF) transmitter to send the captured video, which is then relayed to a receiver connected to either an integrated or cloud-based storage solution. Unlike analog closed-circuit television (CCTV) cameras, network cameras eliminate the dependency on a local recording device, requiring only a local network to capture scenes within a construction site. This innovation not only enhances the efficiency and flexibility of surveillance systems but also underscores the significance of our research in advancing construction site monitoring. Therefore, avoid complex and redundant camera arrangements [49]. The intelligence of camera technology ensures the stability and adaptability of this monitoring terminal. Now, cameras can adapt to light changes and have many intelligent functions, such as auto-focus and light compensation [45].

**Fig 4:**
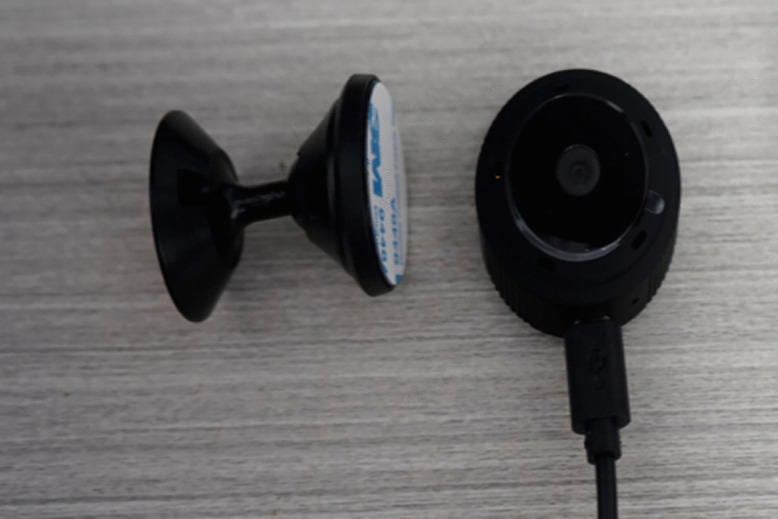
Wireless camera.

This study employed a high-definition camera with a resolution of 3 megapixels (Fig 4). With respect to the utilization of higher-resolution cameras, experimentation with such devices was not pursued owing to financial constraints. Although it is acknowledged that cameras with higher resolution have the potential to yield superior image quality and potentially enhance detection precision, they are accompanied by elevated costs that could compromise the overall feasibility and economy of our system. Consequently, a 3-megapixel camera was selected as an optimal compromise between cost and performance considerations. The camera operates with an internal voltage of DC 5V at 1A and can function within a temperature range of −10 ℃ to 50 ℃. It supports both memory cards and cloud storage for the videos captured. Connectivity is facilitated by WIFI, specifically the IEEE 802.11B/G/N 2.4 GHz standard. The camera, which is cylindrical in shape, measures 4.2 cm in diameter and 1.6 cm in thickness. A key feature is its wireless connectivity, eliminating the need for cumbersome cables. Additionally, the camera is equipped with an integrated web server, enabling it to transmit captured images to the Internet via its Ethernet network interface (10-Base-T/100Base-Tx). This allows for easy access to both static images and videos, consisting of a sequence of images, from any internet-connected computer. The innovative wireless design and advanced connectivity features of this camera system highlight its significance in facilitating remote monitoring and data collection in various research and practical applications.

#### 3.3.2 Wireless network.

The establishment of a wireless local area network (WLAN) is crucial to guarantee timely video transmission. Wireless technologies offer a cost-effective method for connecting various network devices and computers, primarily owing to their straightforward installation process. This aspect is particularly beneficial in construction settings, as it eliminates the potential hazards and operational obstacles posed by physical network cables. By integrating additional components, the bandwidth and coverage area of a WLAN can be significantly enhanced.

The monitoring terminal captures video, which is then transmitted to the server for decoding, viewing, processing, and storage by a computer. In construction sites with intricate wiring conditions, wireless transmission of monitoring video is advantageous due to the ease of assembly and mobility it provides for equipment. This system employs the most widely used wireless network products on the market, taking into account the stability and speed of the WLAN transmission function. Based on the specific environment and requirements of the construction site, appropriate equipment for wireless routers, wireless repeaters, and monitoring server systems is carefully selected. By combining wireless routers and repeaters, the transmission distance of the wireless signal can be tailored to meet our specific needs.

**Fig 5:**
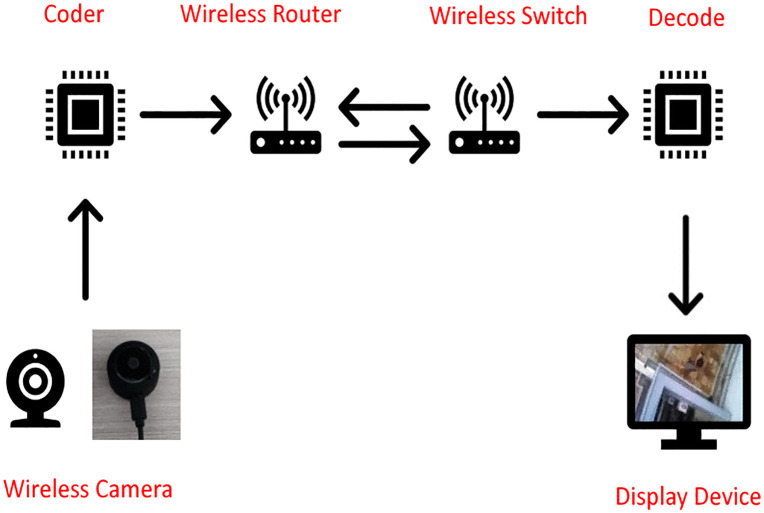
Schematic diagram of the wireless monitoring system.

As illustrated in Fig 5, the camera initially compresses and encodes the video data before transmitting it to the network server via WiFi. The monitoring center accesses this video from the construction site via WiFi and subsequently saves, processes, and analyzes it. This approach underscores the innovation and value of our research, highlighting the significance of wireless technology in enhancing the efficiency and safety of construction site monitoring.

### 3.4 Sub-systems description—Data processing segment

The data processing module is mainly used to realize the automatic detection of workers on the construction site because the accident rate on the construction site is relatively high. The traditional management method of the construction site is usually manual, which is error-prone and cannot detect workers’ violations in time. To solve this problem and improve the efficiency of supervision, the use of computer vision for automatic worker identification will be a good choice. Several studies have focused on the usage of Faster-RCNN to ensure the on-site safety of workers. This algorithm, based on deep learning, is used for non-hardhat-use (NHU) detection [46] or identifying workers and their harnesses [47]. Histogram of oriented gradients (HOG) and Support Vector Machine (SVM) are also used for helmet identification for work safety [48]. To prevent high-altitude falling, a single-shot detector (SSD) based framework for personal protective equipment (PPE) checking and other safety assurance facilities, detecting guardrails like guardrails using VGG16 is developed [49].

This paper adopts an automatic detection algorithm based on YOLO-v5 to address the automatic detection of construction workers. At the same time, the feasibility of the Helikite-based monitoring system can also be reflected indirectly validated by applying the trained model to the collected video. The basic idea of YOLO-v5 target detection is to use regression to get the categories and locations of images. There are five versions before and after this method. Each version is continuously combined with advanced network and detection ideas to improve model performance. The backbone network of YOLO-v5 splits the input photos into an S × S grid. Then each cell is responsible for detecting the target within which the centre point falls. YOLOv5 is mainly divided into four parts: the input side, the backbone network, the neck network, and the prediction. Compared with the previous YOLOv3, these four parts have been optimized differently, and new algorithms have been added to improve the overall performance. Therefore, the article employs YOLOv5 as the object detection network. The selection of YOLOv5 was driven by three primary practical considerations. First, the main objective of this study is to validate the feasibility of the proposed Helikite-based monitoring system, rather than to advance the absolute state‑of‑the‑art in object detection. YOLOv5 is a mature and extensively validated framework that offers an excellent balance between detection accuracy and inference speed, making it a reliable tool for this system-level verification. Second, the long-term goal is to deploy the monitoring capability on embedded edge devices (e.g., NVIDIA Jetson) mounted directly on the Helikite; the mature PyTorch-to-ONNX/TensorRT conversion ecosystem of YOLOv5 provides a practical advantage over many newer architectures that still lack robust deployment optimisation. Third, our experimental platform is constrained to an NVIDIA GTX 1650 GPU, which necessitates a lightweight model to maintain real-time inference. YOLOv5s, with only 7.2 million parameters, meets this requirement while achieving high accuracy.

Beyond these practical motivations, YOLOv5 also offers the following distinct technical advantages:

- It boasts a user-friendly interface and utilizes the PyTorch framework for dataset training, simplifying the generation of weight files compared to the Darknet framework employed by YOLO V4. This enhancement facilitates a more streamlined workflow and reduces computational complexities.
- The tool not only facilitates easy environment configuration but also expedites the model training process. This efficiency is achieved through its support for batch inference, enabling the production of real-time results. This feature significantly enhances the tool’s applicability in time-sensitive scenarios.
- The versatility of this tool extends to its ability to process a variety of input formats, including individual images, batches of images, videos, and even direct feeds from a webcam. This flexibility enhances the tool’s utility across a wide range of applications.
- Furthermore, the weight files generated using PyTorch can be seamlessly converted to the ONNX format, ensuring compatibility with Android devices. Additionally, by utilizing CoreML, these files can be further transformed into a format suitable for integration with OpenCV or iOS, thereby enabling direct implementation into mobile applications. This cross-platform compatibility significantly broadens the potential reach and impact of the tool.

In summary, the presented tool offers a user-friendly interface, efficient training processes, versatile input handling, and cross-platform compatibility, making it a valuable addition to the field of machine learning and computer vision.

## 4. Experiments

To validate the effectiveness of the video footage captured by our system, we intend to employ the recently introduced YOLO-v5 model for precise target detection and subsequent analysis of the recorded visuals. If the achieved accuracy rate satisfies our benchmarks, it would confirm the reliability of our system. In the forthcoming sections, we elaborate on the development process of the worker detection model, highlighting its novelty and significance in enhancing the overall efficiency and accuracy of construction site monitoring.

### 4.1 Data collection

The unique high suspension position of the Helikite offers a distinct overhead, bird’s-eye view perspective for monitoring, facilitating long-range observations. However, a significant challenge arises from the fact that most existing image datasets related to worker identification primarily consist of horizontal or oblique perspectives. Consequently, these datasets are inadequate for Helikite-based monitoring systems aiming to detect and identify workers or equipment captured in the video footage. Therefore, there is a compelling need to develop a specialized dataset featuring a bird’s-eye view of workers and equipment.

**Fig 6:**
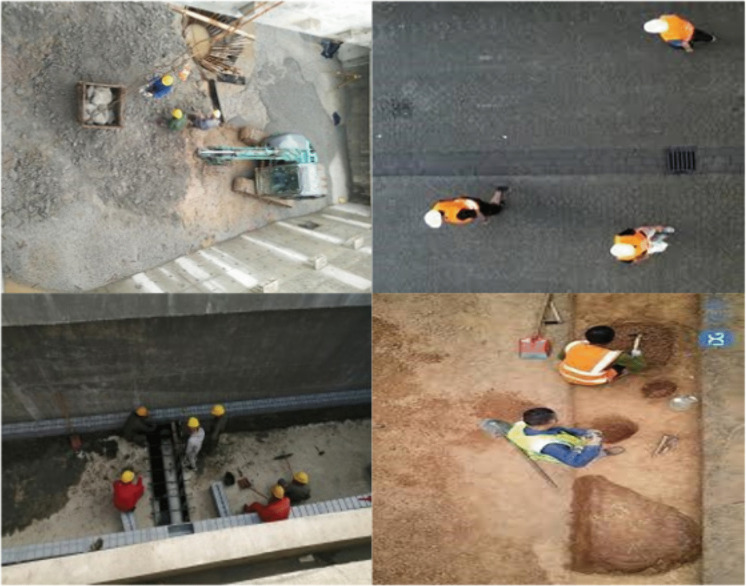
A bird’s eye view of the worker.

Our primary data sources are video monitoring recordings from construction sites, specifically captured using Helikite to hover over workers and equipment. This data was collected at an underground sewage treatment plant situated north of Tianfu Five Street, Chengdu, Sichuan Province, China (No permits were required for this work, as the Helikite operations fell below the 120-meter altitude threshold mandated for national aviation regulatory approval (in accordance with the Civil Aviation Administration of China’s [CAAC] “Regulations on the Administration of Unmanned Aerial Vehicles,” 2021), and the study did not involve protected areas, endangered species, or sensitive information). During data acquisition, the Helikite maintained a flying height of 10 meters above the workers or equipment. Given that construction activities are frequently suspended when wind speeds exceed Level 5 (approximately 8–10 m/s), the present study concentrates on conditions below this threshold. Laboratory tests have disclosed that the Helikite system has an operational wind speed limit of 5 m/s; beyond this, vibrations in the underlying keel compromise data integrity. Notwithstanding this limitation, the Helikite demonstrates notable advantages over unmanned aerial vehicles (UAVs) in windy environments. Firstly, it possesses enhanced endurance, leveraging wind power and helium buoyancy for potentially prolonged airborne durations, whereas UAVs are constrained by battery life. Secondly, the Helikite offers simplified operation, requiring only ground-based control line adjustments, as opposed to the professional remote piloting necessary for UAVs. Thirdly, it exhibits superior stability, with a robust platform design that maintains stability in windy conditions, whereas UAVs are more susceptible to wind disturbances. Consequently, the Helikite system surpasses traditional UAVs in terms of overall performance under windy conditions. A representative sample from this dataset is presented in Fig 6.

The development of this unique dataset addresses a significant gap in the field, enabling more accurate and effective monitoring using Helikite technology. This innovation not only enhances the capabilities of current monitoring systems but also underscores the value and significance of our research in advancing the field of construction site monitoring.

### 4.2 Data enhancement methods

Based on the preceding description, we gathered 1000 images of workers from actual construction sites. To broaden our dataset and bolster the robustness and generalization capability of our model, thereby mitigating the risk of overfitting, we expanded the image count through preprocessing. Furthermore, we employed various data augmentation techniques to enhance worker identification accuracy. These methods encompassed rotation, horizontal flipping, color balance adjustment, brightness alteration, and blurring.

In this study, we opted to utilize Augmentor for data augmentation. Augmentor is a Python library specifically designed for image data augmentation and artificial generation, tailor-made for machine learning projects. Although its primary focus lies in data augmentation, it also incorporates crucial image preprocessing functionalities. Considering the diverse camera angles inherent in the video data acquired through our monitoring approach and the inevitable issue of occlusion, we primarily employed rotation and occlusion for data augmentation. This involved rotations of 90 degrees, −90 degrees, random rotations within the 0–25-degree range, and random occlusions. Ultimately, through data augmentation, we obtained a total of 3000 enhanced images.

The significance of this research lies in its innovative approach to addressing the challenges posed by limited data and variable camera angles in construction site monitoring. By leveraging advanced data augmentation techniques, we have not only expanded our dataset but also ensured the enhanced accuracy and reliability of worker identification. This study paves the way for future research in this domain, highlighting the value and importance of our methodology in the realm of construction site monitoring and beyond.

### 4.3 Image annotation and dataset production

After capturing image frames of workers from a bird’s-eye view, the next crucial step was to annotate them using the graphical annotation tool, LabelImg. These annotations were carefully preserved in an XML format compatible with Python, ensuring ease of access and manipulation. During the curation of the training dataset, all images were resized to a uniform 618x618 pixels, with the width adjusted proportionally to maintain the original aspect ratio. A systematic numbering of images was followed by meticulous manual annotations, involving the precise drawing of bounding boxes and the manual classification of categories.

To mitigate the risk of overfitting in the neural network, positive samples containing inadequate or ambiguous pixel regions were deliberately excluded from the annotation process. Furthermore, in cases of occlusion, we adhered to a strict criterion: targets with an occlusion area exceeding 85% or occupying less than 15% of the image edge area were not annotated. These rigorous measures underscore our commitment to ensuring the highest quality of data for subsequent analysis, highlighting the precision and meticulousness of our methodology.

### 4.4 YOLO-v5 model training progress

The experimental platform employed in this study consisted of a laptop configured with an AMD Ryzen 5 4600H with Radeon Graphics (12 CPUs), a 3.0 GHz processor, 16384 MB RAM, NVIDIA GeForce GTX 1650 graphics card, and a 64-bit Windows 10 operating system. The development environment comprised Python 3.8.11 and PyTorch 1.90. In the training experiments conducted with the YOLOv5 model, a dataset comprising 1000 aerial images of workers was employed as the training set to encompass diverse worker activity scenarios. An additional 100 images were selected to form an independent test set, serving to evaluate the model’s generalization capability. The input images were uniformly resized to 640 × 640 pixels, striking a balance between preserving image details and computational efficiency. During training, a batch size of 4 and a fixed number of 300 iterations were adopted to find the optimal balance between model performance and training duration. The initial learning rate was set at 0.01 to expedite the training process, while a weight decay rate of 0.0005 was implemented to mitigate overfitting. Furthermore, the Intersection over Union (IoU) threshold was established at 0.2, ensuring a reasonable degree of overlap between predicted and ground truth bounding boxes while maintaining an adequate number of positive samples. These parameter settings were meticulously chosen to optimize the YOLOv5 model’s performance for the specific task. Through this training process, an optimal object detection model for the worker detection dataset was obtained.

The results of image recognition are divided into four types of samples [50].

(1) True-positive samples: samples with the predicted result of A and marked as A;
(2) False-positive samples: samples with the predicted result of A and marked as B;
(3) False-negative samples: samples with the predicted result of B and marked as A;
(4) True negative samples: samples whose predicted result is B and whose actual label is also B;

According to the results of the above sample classification, precision and recall rate can be defined. Since TN (true-negative) samples do not affect the results in the experiment, TP (true-positive), FP (false-positive), and FN (false-negative) need to be defined in advance before describing the accuracy, which is further expressed as follows:

(1) True Positives (TP) refer to the count of instances accurately identified as positive cases. Specifically, these are the instances that genuinely represent workers and have been correctly labeled as such by the classifier.
(2) False Positives (FP) denote the number of instances mistakenly categorized as positive cases. In other words, these are instances that are not actually workers but have been erroneously labeled as workers by the classifier.
(3) False Negatives (FN) indicate the number of instances incorrectly labeled as negative. These are instances that are, in fact, workers but have not been identified as such by the classifier.
(4) True Negatives (TN) represent the count of instances accurately identified as negative cases. Specifically, these are instances that are not workers and have been correctly classified as non-workers by the classifier.

Table 1 presents the definitions of TP, FP, FN, and TN.

**Table 1:** Parameter definition of TP, FP, and FN.

Content	Real result (Worker)	Testing result (Worker)
TP	True	True
FP	False	True
FN	True	False
TN	False	False

*Accuracy* represents the ratio of accurate predictions, specifically true positives (TP) and true negatives (TN), to the total number of items evaluated.


$$\begin{array}{c} Accuracy=\frac{TP+TN}{TP+FP+TN+FN} \end{array}$$ (1)

*Precision* is calculated by dividing the number of true positives (TP) by the total count of instances marked as part of the class, which is the combined sum of true positives (TP) and false positives (FP).


$$\begin{array}{c} Precision=\frac{TP}{TP+FP} \end{array}$$ (2)

*Recall* is defined as the ratio of true positives (TP) to the overall count of elements genuinely belonging to the class—specifically, the aggregate of true positives (TP) and false negatives (FN).


$$\begin{array}{c} Recall=\frac{TP}{TP+FN} \end{array}$$ (3)

*F1-score* is determined by both *Precision* and *Recall*.


$$\begin{array}{c} F_{\text{1}}=\frac{\text{2}}{\frac{\text{1}}{Precision}+\frac{\text{1}}{Recall}} \end{array}$$ (4)

## 5. Results

Our main focus was to evaluate the clarity of the captured imagery and the reliability of the system. The wireless camera mounted on the Helikite requires electrical power, and its battery life imposes a constraint on the duration of continuous monitoring. The camera employed in this experiment can sustain uninterrupted transmission for approximately five hours, necessitating battery replacement to maintain monitoring continuity. Furthermore, helium is crucial for maintaining the buoyancy of the Helikite, and any leakage of helium would significantly impair its monitoring capability. The helium balloon used in this study, constructed with an aluminum mold material, ensures leak-free operation for over 24 hours. Consequently, the continuous monitoring time is jointly determined by the camera’s battery capacity and the helium balloon’s floating endurance.

Given the absence of tall buildings surrounding the foundation pit of the sewage treatment plant, coupled with the limited wireless local area network coverage at the construction site, the maximum effective transmission distance was determined to be 20 meters. To ensure optimal performance, the Helikite was maintained within this range. Consequently, we opted to position the aerial monitoring system at an elevation of approximately 10 meters above ground level. The system is capable of achieving a circular monitoring area with a radius of approximately six meters. Although the footage can extend further, excessive distance results in a decline in recognition accuracy, thereby impacting data collection. To enhance the system’s practicality and adaptability to larger construction sites, consideration could be given to utilizing a larger Helikite to carry a higher-pixel camera and placing it at a higher elevation. This approach would serve to expand the monitoring scope while maintaining high precision, thereby better fulfilling the surveillance requirements of large-scale construction sites. Furthermore, considering that construction activities are typically suspended during inclement weather conditions such as rain, snow, and hail, our system is primarily utilized in clear or windy weather.

All images captured by the proposed system were input into YOLOv5 for detection, and evaluate the accuracy of recognition. The comparison is made based on the number of corresponding workers manually counted and the number of tasks identified by the computer. The results are summarized in Table 2.

**Table 2:** Performance metrics of the YOLO-v5 model.

Indicator	*Accuracy*	*Precision*	*Recall*	*F1-score*
Training dataset	0.9836	0.9850	0.9925	0.9801
Validation dataset	0.9827	0.9850	0.9920	0.9820

To evaluate external generalization, the trained model was tested on an independent aerial dataset collected at a separate construction site under different lighting and background conditions. This dataset contains 152 images with manually annotated workers. The model achieved: Accuracy: 0.968; Precision: 0.972; Recall: 0.985; F1-score: 0.978. The result demonstrates robust performance beyond the original training environment.

### 5.1 Statistical validation

Five-fold cross-validation was performed to evaluate the robustness and reproducibility of the worker detection model. The entire dataset was randomly divided into five subsets of approximately equal size. In each iteration, four folds were used for training, and the remaining fold was used for testing, ensuring that each subset was evaluated once.

Performance metrics, including Accuracy, Precision, Recall, and F1-score, were averaged across the five folds. The 95% confidence intervals were computed to quantify statistical uncertainty. The cross-validation results are summarized as follows: Accuracy = 0.981 ± 0.006; Precision = 0.984 ± 0.005; Recall = 0.991 ± 0.004; F1-score = 0.982 ± 0.006. These results demonstrate consistent detection performance across different data splits, indicating that the proposed Helikite-based monitoring system exhibits stable and reliable behavior under varying training–testing configurations.

### 5.2 Computational efficiency and model complexity

To evaluate the practical usability of the proposed system, we further report the computational characteristics of the YOLO-v5 model. The network contains approximately 7.2 million parameters, with a model size of about 14 MB. On the experimental platform equipped with an NVIDIA GTX 1650 GPU, the model achieves an average inference speed of approximately 38 frames per second at an input resolution of 640 × 640. These results indicate that the proposed system satisfies real-time monitoring requirements while preserving high detection accuracy (Precision = 0.9850, Recall = 0.9920). The lightweight architecture and efficient inference of YOLO-v5 support its deployment in practical construction environments and facilitate future integration with edge devices mounted on the Helikite platform.

Nevertheless, this study has not benchmarked YOLOv5 against more recent detectors such as YOLOv10, YOLOv11, or DETR‑based architectures. A direct comparison under our current experimental conditions was not pursued, as these models generally require substantially higher computational resources or rely on customized inference operators that are not yet fully optimised for the edge‑deployment scenarios targeted by the Helikite system. We acknowledge this as a limitation of the present work and plan to conduct a systematic comparison with the latest lightweight object detectors as a key direction for future research, once their on‑device deployment ecosystems have matured sufficiently to guarantee real‑time performance alongside improved accuracy.

## 6. Discussion

### 6.1 Operational performance

Given that our system primarily serves monitoring purposes, a crucial aspect of monitoring involves identifying individuals or objects. Consequently, if the captured video enables target detection with a certain level of accuracy, it signifies the video’s effectiveness. When utilizing the YOLO-v5 model for worker detection from the aerial perspective provided by the Helikite, a notable discrepancy was observed between these bird’s-eye view images and traditional horizontal or oblique view datasets. This divergence suggests that existing worker detection datasets may not be suitable for this unique aerial perspective, a gap also emphasized in experiments, which pointed out that most construction monitoring datasets rely on ground or oblique perspectives, leading to suboptimal performance in aerial scenarios [51]. To address this challenge, the research team collected and annotated a novel dataset tailored specifically for worker detection from the Helikite’s viewpoint.

Furthermore, images captured from an aerial perspective are susceptible to variations in weather conditions, such as changes in illumination, shadows, and fog, as well as the complexity of backgrounds, including construction facilities and ground conditions. These variations can impact image clarity and the performance of the detection model. Additionally, the issue of occlusion significantly affects detection accuracy. On construction sites, occlusion between workers and equipment is a common occurrence. When workers are obscured by other objects, such as scaffolding or tower cranes, the detection performance of the YOLO-v5 model may decline. Although the YOLO-v5 model exhibits a certain level of robustness to occlusion, extreme cases (e.g., occlusion exceeding 85% of the worker’s area) can still compromise detection precision [52].

To mitigate these challenges, severely occluded samples were rigorously excluded during the data annotation process to reduce their interference with model training. Moreover, data augmentation techniques, such as rotation and flipping, were employed to increase sample diversity, thereby alleviating the impact of occlusion on model performance to some extent. Our implemented YOLO-v5 model excels at recognizing workers efficiently, with performance metrics (validation precision = 0.9850, recall = 0.9920) highlighting the effectiveness of both our dataset and the Helikite-captured imagery.

### 6.2 Quantitative comparison with UAV-based systems

Recent studies have demonstrated the effectiveness of UAV platforms for construction-site monitoring; however, reported flight durations typically range from 20 to 40 minutes per battery cycle [53], with operational wind tolerance generally below 5 m/s and deployment costs commonly lower than high-end drones like the DJI Mavic 3 Pro. In contrast, the proposed Helikite-based system enables continuous aerial observation exceeding 12 hours, primarily limited by camera battery capacity, while maintaining stable operation at wind speeds up to approximately 5 m/s. The total hardware cost of the Helikite platform is approximately USD 600, including the aerostat, tethering system, and imaging sensor.

A quantitative comparison between representative UAV-based monitoring systems reported in recent literature and the proposed Helikite platform is summarized in Table 3.

**Table 3:** Quantitative comparison between UAV-based systems and Helikite platform.

Platform	Endurance	Wind tolerance	Payload	Deployment cost	Monitoring mode
UAV-based systems	25–40 min	<5 m/s	0.5–2 kg	USD 2,000–8,000	Mobile
Proposed Helikite	>12 h	~5 m/s	~1 kg	~USD 600	Fixed-point

Although UAVs provide superior spatial mobility and rapid area coverage, their short endurance and frequent battery replacement constrain their suitability for persistent monitoring. By exploiting combined helium buoyancy and aerodynamic lift, the Helikite system offers long-duration, fixed-point aerial observation with significantly reduced operational cost. This establishes a clear application niche: UAV platforms are better suited for short-term mobile inspection, whereas the Helikite platform is optimized for continuous, low-cost, stationary monitoring of construction activities.

## 7. Limitations and future directions

This study demonstrates the potential of the Helikite system, yet it also reveals operational limitations that frame the scope for future research. The primary constraint is wind speed. As identified in our experiments, keel vibrations begin to compromise data integrity at wind speeds exceeding 5 m/s. Although this operational limit is already superior to many UAVs in windy conditions and aligns with common work stoppages (at Level 5 winds, ~ 8–10 m/s), enhancing the system’s wind resistance is a critical next step. Future work will focus on optimizing the Helikite’s aerodynamic design: (1) redesigning the kite wing structure using lightweight, high-strength composite materials to enhance lift and stability in winds exceeding 5 m/s, and (2) integrating a micro-stabilization platform (weight < 500g) to actively counteract keel vibrations, ensuring image clarity at higher wind speeds. Prototype testing will be conducted in controlled wind tunnel environments to validate performance under 8–10 m/s conditions.

Furthermore, the dusty environment of construction sites can lead to progressive image degradation. To mitigate this, the camera module will be upgraded with: (1) a self-cleaning hydrophobic lens coating to repel dust particles, and (2) an automated air blower system activated by a dust sensor (sensitivity: > 10 particles/mm²) to remove accumulated debris. Field tests in dusty construction zones will be conducted to verify sustained image quality over 72-hour periods.

Finally, the system’s continuous operational duration is constrained by the camera’s battery life and helium leakage. To address this, we propose two technical solutions: (1) replacing the current camera battery with a high-capacity lithium-polymer cell (10,000 mAh) to extend continuous monitoring to 12 hours, and (2) developing a helium replenishment system—a small, portable pump connected to the balloon via a secondary tether—to compensate for leakage (target: < 0.2%/hour). These upgrades will be validated through 48-hour continuous operation trials.

Looking beyond these technical refinements, future research will explore the integration of a multi-sensor array (e.g., thermal imaging cameras, ultrasonic rangefinders) to enable equipment tracking and proximity hazard detection. Additionally, integrating the system with a cloud-based data fusion platform to synchronize Helikite-captured data with BIM models will facilitate real-time progress monitoring and digital twin applications, further enhancing its value for construction management.

## 8. Conclusion

Traditional monitoring systems relying on fixed cameras have long been used on construction sites, but often suffer from occlusion, especially in dynamic and rapidly evolving construction environments. To address this, this study presented an aerial monitoring platform that innovatively merged a tethered balloon with a kite, forming a Helikite-based monitoring system. The study detailed the meticulous design and rigorous testing processes of this Helikite-based system, which integrated a wireless camera and data transmission system to offer flexible aerial monitoring of construction sites with adjustable positioning. The reliability of the video data captured by the system was verified using the YOLO-V5 model for worker recognition, achieving an accuracy rate exceeding 98% on the validation set. This result indirectly confirmed the system’s suitability for construction site monitoring, highlighting its efficacy and reliability. In conclusion, the Helikite-based construction monitoring system emerges as a mobile, wide coverage, and cost-effective solution. Its versatility and performance significantly contribute to enhancing construction management, paving the way for safer, more efficient construction practices.

## Supporting information

S1 File Helikite construction monitoring. (ZIP)

## References

[1] Guo S, Xiong C, Gong P. A real-time control approach based on intelligent video surveillance for violations by construction workers. J Civil Eng Manag. 2018;24(1):67–78. doi: 10.3846/jcem.2018.301

[2] Leung S, Mak S, Lee BLP. Using a real-time integrated communication system to monitor the progress and quality of construction works. Autom Constr. 2008;17:749–57.

[3] Cheng T, Teizer J. Real-time resource location data collection and visualization technology for construction safety and activity monitoring applications. Autom Construct. 2013;34:3–15. doi: 10.1016/j.autcon.2012.10.017

[4] Kim J, Ham Y, Chung Y, Chi S. Systematic camera placement framework for operation-level visual monitoring on construction jobsites. J Constr Eng Manag. 2019;145(04019019). doi: 10.1061/(asce)co.1943-7862.0001636

[5] Tomé A, Kuipers M, Pinheiro T, Nunes M, Heitor T. Space–use analysis through computer vision. Automation in Construction. 2015;57:80–97. doi: 10.1016/j.autcon.2015.04.013

[6] Kim D, Liu M, Lee S, Kamat VR. Remote proximity monitoring between mobile construction resources using camera-mounted UAVs. Automat Construct. 2019;99:168–82. doi: 10.1016/j.autcon.2018.12.014

[7] Gong J, Caldas CH, Gordon C. Learning and classifying actions of construction workers and equipment using bag-of-video-feature-words and bayesian network models. Adv Eng Inform. 2011;25(4):771–82. doi: 10.1016/j.aei.2011.06.002

[8] Seo J, Han S, Lee S, Kim H. Computer vision techniques for construction safety and health monitoring. Adv Eng Inform. 2015;29(2):239–51. doi: 10.1016/j.aei.2015.02.001

[9] Yang J, Shi Z, Wu Z. Vision-based action recognition of construction workers using dense trajectories. Adv Eng Informatics. 2016;30:327–36. doi: 10.1016/j.aei.2016.04.009

[10] Heyns AM, du Plessis W, Curtin KM, Kosch M, Hough G. Analysis and exploitation of landforms for improved optimisation of camera-based wildfire detection systems. Fire Technol. 2021;57:2269–303. doi: 10.1007/s10694-021-01120-2

[11] Heyns A, du Plessis W, Kosch M, Hough G. Optimisation of tower site locations for camera-based wildfire detection systems. Inter J Wildland Fire. 2019;28(9):651–65. doi: 10.1071/wf18196

[12] Zhao J, Yoshida R, Cheung SS, Haws D. Approximate Techniques in Solving Optimal Camera Placement Problems. International Journal of Distributed Sensor Networks. 2013;9(11):241913. doi: 10.1155/2013/241913

[13] Tian W, Li H, Zhu H, Wang Y, Liu X, Yang R, et al. A review of smart camera sensor placement in construction. Buildings. 2024;14(12):3930. doi: 10.3390/buildings14123930

[14] Chen X, Zhu Y, Chen H, Ouyang Y, Luo X, Wu X. BIM-based optimization of camera placement for indoor construction monitoring considering the construction schedule. Automat Construct. 2021;130:103825. doi: 10.1016/j.autcon.2021.103825

[15] Asadi K, Kalkunte Suresh A, Ender A, Gotad S, Maniyar S, Anand S, et al. An integrated UGV-UAV system for construction site data collection. Autom Constr. 2020;112:103068. doi: 10.1016/j.autcon.2019.103068

[16] Lee JH, Park J-H, Jang B-T. Design of robot based work progress monitoring system for the building construction site. In: 2018 International Conference on Information and Communication Technology Convergence (ICTC), 2018. 1420–2. 10.1109/ictc.2018.8539444

[17] Zhang J, Campbell JF, Sweeney DC, Hupman AC. Energy consumption models for delivery drones: a comparison and assessment. Transp Res Part D Transp Environ. 2021;90. doi: 10.1016/j.trd.2020.102668

[18] Liang H, Lee S-C, Bae W, Kim J, Seo S. Towards UAVs in construction: advancements, challenges, and future directions for monitoring and inspection. Drones. 2023;7(3):202. doi: 10.3390/drones7030202

[19] Han D, Lee S, Song M, Cho J. Change detection in unmanned aerial vehicle images for progress monitoring of road construction. Buildings. 2021;11(4):150. doi: 10.3390/buildings11040150

[20] Kung RY, Pan NH, Wang CCN, Lee PC. Application of deep learning and unmanned aerial vehicle on building maintenance. Advances in Civil Engineering. 2021;2021:5598690. doi: 10.1155/2021/5598690

[21] Mahajan G. Applications of drone technology in construction industry: A study 2012-2021. Int J Eng Adv Technol. 2021;11:224–39.

[22] Hoseini SA, Hassan J, Bokani A. In situ MIMO-WPT recharging of UAVs using intelligent flying energy sources. 2021. 1–15.

[23] Gong A, Verstraete D. Fuel cell propulsion in small fixed-wing unmanned aerial vehicles: current status and research needs. International J Hydrogen Energy. 2017;42(33):21311–33. doi: 10.1016/j.ijhydene.2017.06.148

[24] Alioto ZA. Design of a solar assisted flying wing small unmanned aerial system. 2022.

[25] Shen J, Wang B, Chen BM, Bu R, Jin B. Review on wind resistance for quadrotor UAVs: modeling and controller design. Unmanned Syst. 2023;11: 5–15.

[26] Gao M, Hugenholtz CH, Fox TA, Kucharczyk M, Barchyn TE, Nesbit PR. Weather constraints on global drone flyability. Sci Rep. 2021;11(1):12092. doi: 10.1038/s41598-021-91325-w 34103585 PMC8187708

[27] Akcesme G. Naval Postgraduate. 2014.

[28] Aglietti GS. Dynamic response of a high-altitude tethered balloon system. J Aircr. 2009;46:2032–40. doi: 10.2514/1.43332

[29] Lambert C, Saunders A. Design of a one-third scale multi-tethered aerostat system for precise positioning of a radio telescope receiver. CASI Flight Mech. 2003;1–12.

[30] Bo W, Dengfeng D, Zhizhong Z, Weihu Z. Research on stabilized tracking turntable based on balloon tethered by ship. In: 2019 14th IEEE International Conference on Electronic Measurement and Instruments (ICEMI), 2019. 482–8. 10.1109/icemi46757.2019.9101729

[31] Zhang W. The integrated panoramic surveillance system based on tethered balloon. In: IEEE Aerosp. Conf. Proc., 2015. 1–7. 10.1109/AERO.2015.7118948

[32] Alsamhi SH, Samar Ansari M, Rajput NS. Disaster coverage predication for the emerging tethered balloon technology: capability for preparedness, detection, mitigation, and response. Disaster Med Public Health Prep. 2018;12(2):222–31. doi: 10.1017/dmp.2017.54 28789726

[33] Liao L, Pasternak I. A review of airship structural research and development. Prog Aerosp Sci. 2009;45:83–96. doi: 10.1016/j.paerosci.2009.03.001

[34] Mahmood K, Ismail NA, Suhadis NM. Tethered aerostat envelope design and applications: A review. In: AIP Conference Proceedings, 2020. 050003. 10.1063/5.0002358

[35] Cheng S. Tethered balloon system for video surveillance. Sci Technol Eng. 2011;11:7991–4.

[36] Status D, Balloon AT. Analysis on developing status and trend of American tethered balloon - borne early warning system. 2010.

[37] Wang Z, Huang M, Han W, Zhao B, Zhang G, Qian L, et al. Optical sensing in Tibet Plateau wildlife observation based on tethered balloon. Optik. 2021;243:167425. doi: 10.1016/j.ijleo.2021.167425

[38] Pang C, He Z, Song K, Cao S. Analysis of wind field response characteristics of tethered balloon systems. Aerospace. 2024;11(5):360. doi: 10.3390/aerospace11050360

[39] Le V, Doulgeris KM, Komppula M, Backman J, Bagheri G, Bodenschatz E, et al. Dataset of airborne measurements of aerosol, cloud droplets and meteorology by tethered balloon during PaCE 2022. Earth Syst Sci Data Discuss. 2025;2025:1–19.

[40] Alsamhi SH, Gupta SK, Rajput NS. Performance evaluation of broadband service delivery via tethered balloon technology. In: 2016 11th International Conference on Industrial and Information Systems (ICIIS), 2016. 133–8. 10.1109/iciinfs.2016.8262921

[41] Chandrasekharan S, Gomez K, Al-Hourani A, Kandeepan S, Rasheed T, Goratti L, et al. Designing and implementing future aerial communication networks. IEEE Commun Mag. 2016;54(5):26–34. doi: 10.1109/mcom.2016.7470932

[42] Bucaille I, Hethuin S, Munari A, Hermenier R, Rasheed T, Allsopp S. Rapidly deployable network for tactical applications: aerial base station with opportunistic links for unattended and temporary events ABSOLUTE Example. In: MILCOM 2013 - 2013 IEEE Military Communications Conference, 2013. 1116–20. 10.1109/milcom.2013.192

[43] Verhoeven GJJ, Loenders J, Vermeulen F, Docter R. Helikite aerial photography - a versatile means of unmanned, radio controlled, low-altitude aerial archaeology. Archaeol Prospect. 2009;16(2):125–38. doi: 10.1002/arp.353

[44] Chandrasekharan S, Gomez K, Al-Hourani A, Kandeepan S, Rasheed T, Goratti L, et al. Designing and implementing future aerial communication networks. IEEE Commun Mag. 2016;54(5):26–34. doi: 10.1109/mcom.2016.7470932

[45] Zhou J, Cui W, Zhang T. Research on adaptive adjustment technology of intelligent photoelectric detection system. In: 2019 Int. Conf. High Perform. Big Data Intell. Syst. HPBD IS 2019, 2019. 132–5. 10.1109/HPBDIS.2019.8735463

[46] Fang Q, Li H, Luo X, Ding L, Luo H, Rose TM, et al. Detecting non-hardhat-use by a deep learning method from far-field surveillance videos. Autom Constr. 2018;85:1–9. doi: 10.1016/j.autcon.2017.09.018

[47] Fang W, Ding L, Luo H, Love PED. Falls from heights: a computer vision-based approach for safety harness detection. Autom Constr. 2018;91:53–61. doi: 10.1016/j.autcon.2018.02.018

[48] Wu H, Zhao J. An intelligent vision-based approach for helmet identification for work safety. Comput Ind. 2018;100:267–77. doi: 10.1016/j.compind.2018.03.037

[49] Fang Q, Li H, Luo X, Ding L, Luo H, Li C. Computer vision aided inspection on falling prevention measures for steeplejacks in an aerial environment. Autom Construction. 2018;93:148–64. doi: 10.1016/j.autcon.2018.05.022

[50] Jude Chukwura Obi. A comparative study of several classification metrics and their performances on data. World J Adv Eng Technol Sci. 2023;8(1):308–14. doi: 10.30574/wjaets.2023.8.1.0054

[51] Tsai YS, Sit YH. Aerial object tracking with attention mechanisms: accurate motion path estimation under moving camera perspectives. C Model Eng Sci. 2025;143:3065–90.

[52] Lu F, Yao S, Sun G, Zhou T, Huang Y. FMS-YOLO: a lightweight safety belt detection algorithm for high-altitude workers based on attention mechanism and efficient architecture. J Real-Time Image Proc. 2025;22(2). doi: 10.1007/s11554-025-01669-z

[53] Khan M, Qureshi I, Khanzada F. A Hybrid communication scheme for efficient and low-cost deployment of future flying ad-hoc network (FANET). Drones. 2019;3(1):16. doi: 10.3390/drones3010016

